# Patient and caregiver use of patient portal features in primary care: a cross-sectional survey study of Ontarians

**DOI:** 10.1017/S1463423626101376

**Published:** 2026-06-19

**Authors:** Alexis Aomreore, Simone Dahrouge, Kiran Saluja, Simon Lam, Rachelle Ashcroft

**Affiliations:** 1 C.T. Lamont Primary Health Care Research Centre, Bruyere Health Research Institute, Ottawa, Canada; 2 C.T. Lamont Primary Health Care Research Centre; Department of Family Medicine, Bruyere Health Research Institute; University of Ottawa, Ottawa, Canada; 3 Factor-Inwentash Faculty of Social Work, University of Toronto, Toronto, Canada

**Keywords:** asynchronous care, caregivers, patient portals, primary care, patients, virtual care

## Abstract

**Background:**

Patient portals (PPs) are digital healthcare programs providing patients with access to their electronic health records with features aiding patients in managing their care. Although PPs are now widely used in primary care (PC), the desirability of its features is less known.

**Aim:**

This study aims to describe the current and anticipated use of PP features and the socio-demographic profile of PP users accessing these features.

**Methods:**

A cross-sectional survey of patient and caregiver experiences with virtual care in PC in Ontario, Canada, was conducted from December 2022 to March 2023. A survey section captured use/anticipated use of PP features: ‘communicating with practice’, ‘viewing records’, ‘entering record information’, ‘receiving practice information’, and ‘scheduling appointments’. We report on respondents with/without PP access and their use/anticipated use of PP features, and analyzed associations between respondent socio-demographic data and the current use of a number of PP features using Pearson chi-square test.

**Findings:**

A total of 970 responses (743 patients (P) and 227 caregivers (C)) were obtained with respondents having PP access (P:57%; C:62%) and indicating using all (P: 43%; C:45%), some (P:55%; C:52%), or no (P: 2%; C:4%) PP features. For those with PP access, age (65+ years (14%) compared to <65 years (1%)), employment status (unemployed (6%) compared to employed (1%)), and language at home (non-English (9%) compared to English (1%)) were significantly associated with not using any PP features (percentages represent those not using any PP feature). Our findings provide insights into equity aspects to be considered for future PP implementation.

## Introduction

### Brief introduction to patient portals (PPs)

The emergence of the COVID-19 pandemic triggered the need to conduct healthcare visits virtually, by telephone or video (Majeed *et al*., 2020; Glazier *et al*., 2021). At the same time, the use of patient portals (PPs) through which asynchronous care (communication of health information between patients and providers non-simultaneously (Culmer *et al*., 2023)) can be provided and which had already penetrated hospitals began to take a foothold across a larger segment of the healthcare sector (Canadian Institute of Health Information, 2023; Leighton *et al*., 2024).

PP is a generic term referring to structures that allow patients to access some features of care that are typically only available through in-person visits at the practice (Irizarry *et al*., 2015; Gorfinkel and Lexchin, 2019; Ontario Health, 2021; Office of the National Coordinator, no date). The available PP features vary considerably and include the ability to schedule appointments; view chart information such as blood pressure measurements; request prescription renewals; view blood test, CT or MRI scan and imaging results; update health information on PP platform; and allow asynchronous communication with providers (Masterman *et al*., 2016; Lafata *et al*., 2018; Dendere *et al*., 2019; Casacchia *et al*., 2022). At the time of the pandemic, majority of hospitals had adopted PPs, but this was not the case in primary care (PC).

### PPs in primary care globally and in Canada

Prior to the pandemic, the use of PPs in PC was limited in North America but had already gained a good foothold in the United Kingdom since 2015 (Gorfinkel and Lexchin, 2019; NHS England, 2019; Strawley and Richwine, 2023). A meta-analysis of PPs in PC found patients in the United States and Europe to have a PP adoption rate of 52% (Fraccaro *et al*., 2017); however, a much lower adoption rate of 20% was observed in Canada in a different meta-analysis (Hagens *et al*., 2020). The low adoption rate could be the result of inconsistent PP access (El-Toukhy *et al*., 2020), minimal physician experience with PPs (Powell and Myers, 2018), physician concerns regarding patient misinterpretation of test results (Maybee and Greenberg, 2019), or patient’s lack of digital skills (Alkir-Yurt *et al*., 2023). However, patient demand for access to their health records persists and has increased interest in PPs in PC in Canada (Gorfinkel and Lexchin, 2018).

Over the past decade, discussions regarding the implementation of PPs in Canada have resulted in provincial and health system initiatives being established to facilitate PP implementation and increase PP adoption (Hagens, 2023). The Canadian government provided a recommendation to include PP management in physician fee schedules (Canadian Medical Association, 2020) and the Ontario government developed a PP implementation guide (Ontario Health, 2021) to facilitate PP implementation and adoption in PC during the pandemic. The pandemic also increased demand for PPs as the percentage of Canadians using PPs to request prescription renewals and electronically access personal health information increased from 12% and 20% in 2019 to 20% and 27% in 2020, respectively (Canada Health Infoway, 2020).

### Impact of PPs in primary care

The impact of using PPs in PC has been well established. Patients report positive experiences with PPs as they reduce potential exposure to illness by reducing the need for an in-person visit and allow patients to save money and time on transport to appointments (Canada Health Infoway, 2020). PPs serve a pivotal role in care delivery by fostering enhanced patient engagement, and improving patient–provider relationship and patient health outcomes (Carini *et al*., 2021; Han *et al*., 2019). Patient satisfaction scores associated with PP use tend to be high (Crockett and Carter-Templeton, 2020; Lee *et al*., 2022), and studies show that PPs facilitate improved communication between patients and their providers (Apker *et al*., 2021; Lee *et al*., 2022). PC providers also report benefits associated with PP adoption as they facilitate shared information with other health providers in their patients circle of care and enhance communication with their patients (Miller *et al*., 2016; Elers and Nelson, 2018; Zhong *et al*., 2020).

### Objectives

Previous studies have explored the adoption of PPs in PC and found that PP users in PC tend to be younger, White, and have higher number of comorbidities (Lafata *et al*., 2018; Casacchia *et al*., 2022). However, studies have not assessed the anticipated use of PP features, or the socio-demographic profile of individuals currently using PP features in PC post-pandemic. This report stems from a larger research study that aimed to assess patient and caregiver experiences with virtual care in Ontario, Canada (Ashcroft *et al*., 2023). In the larger study, consultations with stakeholders led us to include asynchronous care and to include questions to capture use and anticipated use of PP features. Due to the increasing and consistent use of PPs in virtual care, we conducted this sub-study to explore the use and anticipated use of PP features in depth. The objective of this study is to describe the current and anticipated use of specific PP features and to describe the socio-demographic profile of individuals using a number of specific PP features in Ontario, Canada. An understanding of PP use and user profile post-pandemic can facilitate refined government policies on the use of PPs in delivering healthcare as well as provide insight on tailoring PPs to the needs of the population.

## Methods

### Study design

A population-based, comprehensive, electronic cross-sectional survey of adult patient and caregiver experiences with virtual care in PC was conducted and we report here on PP usage. The survey was offered in both English and French.

### Data collection

The data used in this study are based on a small section of a larger survey which was adapted from our earlier 2021 survey (Ashcroft *et al*., 2023). Research team members, members of the study patient advisory committee and knowledge users pretested the survey. The survey was pretested to ensure that the questions were clear and to ensure readability and format consistency on various devices (e.g., laptop, computer, and phone). Feedback received on the relevant survey sections involved the addition of the patient portal definition in Q9.8 and including ‘check all that apply’ in Q11.2 (Appendix B).

Four research team members, six individuals from the patient advisory committee and knowledge users were involved in pretesting the survey: eight identified as women, six were 55+ years of age and three individuals were from Toronto, four from Ottawa, two from Northern Ontario and one from Southern Ontario. The questionnaire sections used in this study are shown in Appendix B.

#### Data elements

Respondents were required to answer the consent question (Q1.2 in Appendix B) before proceeding with the survey. The patient questionnaire captured the individual’s socio-demographic information and the presence of chronic conditions, while the caregiver questionnaire captured the same items and elicited the profile of the individuals for whom they provided care. Socio-demographic profiling included age, gender, sexual orientation, language spoken at home, marital status, education level, employment status, household income level, immigration status, living situation, race, location, and chronic conditions.

The survey provided a layman’s definition of PPs, and respondents were asked if their PC practice offered access to a PP. If they did, they were directed to answer questions about their utilization of its different features. If they did not, they were asked about their anticipated use of these features. Five common features were assessed: whether they were able to schedule appointments online, view chart information, enter information into chart, communicate with the practice, and receive documents from their practice. Response options were yes (uses/would use), no (does not/would not use), and feature not offered/not sure.

### Eligibility criteria

Ontario patients, or the caregiver of a patient, who had at least one virtual appointment with a PC provider in the past 12 months were eligible to complete the survey. Caregivers completed the survey on behalf of the patient for whom they provided care. Respondents were required to be 18 years of age or older.

Respondents had to have a virtual visit to complete the assessment of their virtual care experience. There were responses from respondents who did not have a virtual visit, and these were excluded from sections in the larger survey assessing virtual care experiences (experience with telephone and video modalities). However, these were retained in other sections of the survey such as the PP section of the survey.

### Survey administration and study preparation

The survey was conducted in Ontario, Canada from December 2022 to March 2023 using Qualtrics. A multipronged approach to recruitment was utilized, and we offered a $5 gift card compensation to each respondent who completed the survey. The study was advertised in English and French on various social media platforms, we reached out to English and Francophone PC clinics via email and sent recruitment materials via email to 17 patient advocacy groups, 75 cultural groups, and 25 health advocacy groups. A patient advisory committee and knowledge users were also engaged to disseminate the recruitment materials (including survey links) to their networks. Two PC providers who were knowledge users sent the recruitment materials to their patients, and the patient advisory committee invited health organizations of which they were members to circulate these to their memberships.

### Ethical consideration

The study was approved by the University of Toronto ethics board (43445 & 43446), and the Bruyere Research Institute (M16-22-054 & M16-22-055). Survey responses were anonymous, and the survey data was kept in a secure password protected OneDrive institutional account on the University of Toronto server.

### Statistical analysis

#### Data cleaning

A honeypot question (University of Toronto, 2024) was included at the start of the survey to prevent bot responses. However, the wide dissemination of the survey link and the $5 gift card compensation contributed to the influx of bot responses which penetrated the preventive measures built into the survey design. All text-option survey responses were manually reviewed to determine if the respondent met the criteria to be excluded as ‘confirmed bots’, retained as ‘potential bots’, or retained as ‘verified responses’. Surveys that were completed in less than 5 minutes, contained responses in languages other than English or French, or contained implausible responses in comment boxes (i.e. such as referring to virtual dating and law), were deemed to be ‘confirmed bots’ and were excluded. Records in which comments were repeated verbatim across multiple sequential records, had questionable comments related to nursing, teaching or learning modes, had a postal code not matching the indicated city, or had suspicious email addresses where the email was not similar to the respondents’ name were deemed ‘potential bots’ and were retained in the sensitivity analysis. Responses which did not meet the criteria for ‘confirmed bots’ or ‘potential bots’ were considered ‘verified responses’ and included in the main analysis. A full description of this process can be found in our 2023 report (Ashcroft *et al*., 2023).

#### Descriptives

The socio-demographic profile of the participants, their access to PPs, and their use/anticipated use of PP features were analyzed descriptively for patients and caregivers separately using percentages. One question asked if the respondents practice offered a PP, and we report this as access to a PP. Race, location and chronic condition responses were categorized as follows: race into White, Indigenous and Other (non-White or non-Indigenous), location into the six Ontario health regions (Toronto, Central, East, West, North East, and North West) (Ontario Health, 2022), and chronic conditions to indicate the number of chronic conditions a respondent indicated they had. Those who indicated ‘prefer not to answer’ to socio-demographic questions and indicated, ‘not offered/not sure’ to use/anticipated use of PP feature questions were regarded as ‘missing data’ and removed from the denominator when calculating the percentages.

Respondents’ use/anticipated use of PP features were grouped into those who use/would use all five, some (1-–4), or none of the PP features. It was difficult to determine if respondents with access to PPs indicated ‘not sure or not offered’ as their practice not offering the feature. This would mean that if a respondent indicated ‘yes’ to four of the five features and indicated ‘not sure or not offered’ in one feature, it could be interpreted as using some features (4 out of 5 features) or using all features (4 out of 4 features) where the four features are the only features offered by the practice. Therefore, for respondents with PP access, we derived the categories by calculating a numerator (i.e., a sum of the number of features they indicated use for), calculating a denominator (i.e., a sum of the number of features they indicated use or don’t use for), and dividing the numerator by the denominator to provide a value, i.e., 1 = use all features; 0 = use no features; any number in between 0 and 1 = use some features. Missing data was excluded from all percentage calculations for all variables.

In addition to the responses received from the broad dissemination of the survey, responses were received from two PC practices. Responses from these practices were weighed to represent 10 patients to prevent errors due to intra-cluster correlation.

#### Associations

To assess associations between socio-demographic factors and the number of PP features currently used, we carried out a Pearson chi-square. Combining the patient and caregiver results, we report on the statistically significant (*p* < 0.05) percentages of those who currently use all, some, and none of the PP features. All analyses were carried out using the IBM SPSS Statistics software (version 29.0.1.0).

#### Sensitivity analysis

The sensitivity analyses were carried out including the verified and bot responses, and we report on our verified results and indicate if we observe variations between the verified and sensitivity results that were 10% or more.

## Results

Among 3019 respondents, 521 records which did not complete any sections of the survey and 169 confirmed bots were excluded. Of the remaining 2329, 1758 completed the section of interest consisting of 970 verified responses and 788 potential bots responses. The results here are based on the 970 verified responses.

Most patient respondents (P: 64%) but only a third of the patients for whom the caregivers responded (C: 34%) were younger than 45 years. These groups were distinct in their marital status (single - P: 35%, C: 44%), education level (<university degree - P: 52%; C: 76%), and household income (<than $50k – P: 23%; C: 45%). The socio-demographic profile of respondents is shown in Table 1.

**Table 1.** Socio-demographic profile of participants Table 1 long description.

Category	Respondents	Patients n (%) (*n* = 743)	Caregivers *n* (%) (*n* = 227)^1^
**Socio-demographic factors**			
Age (*P* = 20; C = 29)	44 and younger	465 (64)	67 (34)
	45 – 65	196 (27)	85 (43)
	65 and older	63 (9)	46 (23)
Gender (*P* = 20; C = 14)	Male	321 (44)	115 (54)
	Female	388 (54)	91 (43)
	Other	14 (2)	7 (3)
Sexual orientation (*P* = 20; C = 19)	Queer	135 (19)	12 (6)
Language spoken at home (*P* = 18; C = 16)	Non-English^2^	76 (10)	27 (13)
Marital status (*P* = 22; C = 22)	Single^3^	251 (35)	91 (44)
Education level (*P* = 25; C = 21)	Less than a university degree	379 (52)	153 (76)
Employment (*P* = 21; C = 24)	Not employed^4^	146 (19)	102 (49)
Household income (*P* = 42; C = 63)	Less than $50k	153 (23)	84 (45)
Immigration (*P* = 41; C = 54)	Recent Immigrant^5^	65 (9)	19 (10)
Lives alone (*P* = 37; C = 51)	Yes	140 (20)	46 (24)
Race/ethnicity* (*P* = 23; C = 15)	White	498 (69)	134 (63)
	Indigenous	69 (10)	25 (12)
	Other	153 (21)	53 (25)
Ontario health region (*P* = 108; C = 51)	Toronto	179 (28)	62 (35)
	Central	170 (27)	41 (23)
	East	105 (17)	30 (17)
	West	128 (20)	35 (20)
	North East	37 (6)	6 (3)
	North West	16 (3)	2 (1)
**Health-related factors**			
Chronic conditions (*P* = 13; C = 4)	Median (Q1, Q3)**	1.0 (0.0, 2.0)	2.0 (1.0, 3.0)
	Three or more conditions (%)	139 (19)	75 (34)

The number of missing respondents is indicated in brackets in each category. Note: the missing data contain both respondents who indicated that they ‘prefer not to answer’ and respondents with missing responses.

1 Represents the profile of the patients on behalf of whom the caregivers responded.

2 Includes French and other non-English languages (respondents were able to select more than one language).

3 Single = never legally married, separated but still legally married, divorced, and widowed.

4 Not Working = not employed/looking for work, unable to work due to sickness or disability, retired from paid work, in school, looking after home/family.

5 Recent Immigrant = Individual who immigrated to Canada in past 10 years.

* Respondents could select more than one option regarding their race. Other includes Black, Arab, Chinese, Filipino, Japanese, Korean, Latin American, South Asian, Southeast Asian, West Asian.

** Q1 – first quartile; Q3 – third quartile.

### Patient portal access and use/anticipated use of PP features and use of a number of PP features

Most respondents indicated having access to a PP (P: 57%; C: 62%). Overall, between 60–73% of patients and 60–74% of caregivers used PPs for communication with their practice, viewing and entering information into their chart, receiving documents from their providers, and scheduling appointments. Between 70–75% of patient and 45–58% of caregiver respondents without current access to PPs (P: 43%; C: 38%) reported that they would use the various features of PP as shown in Table 2.

**Table 2.** Current/anticipated use of features for respondents with and without access to PPs Table 2 long description.

	Current use of PP features* *n* (%)		Anticipated use of PP features** *n* (%)	
	Patients (*n* = 426)	Caregivers (*n* = 140)	Patients (*n* = 317)	Caregivers (*n* = 87)
Schedule appointments online	278 (66)	93 (70)	218 (74)	43 (52)
View chart information	276 (65)	86 (64)	224 (75)	41 (50)
Enter information into chart	250 (60)	80 (60)	210 (71)	47 (56)
Communicate with practice	308 (73)	102 (74)	207 (70)	49 (58)
Receive documents from practice	280 (67)	96 (72)	216 (74)	38 (45)

*For those with PP access; **For those without PP access.

Respondents with access to PP indicated that they currently use all the five listed PP features (P: 43%; C: 45%), use some features (P:55%; C:52%), and use no features (P: 2%; C: 4%). Respondents without access to PPs report that they will use all listed PP features (P:46%; C: 20%), will use some features (P: 42%; C: 72%), and will not use any features (P: 11%; C: 8%).

### Associations between the number of PP features currently used and socio-demographic factors

**Figure 1.**
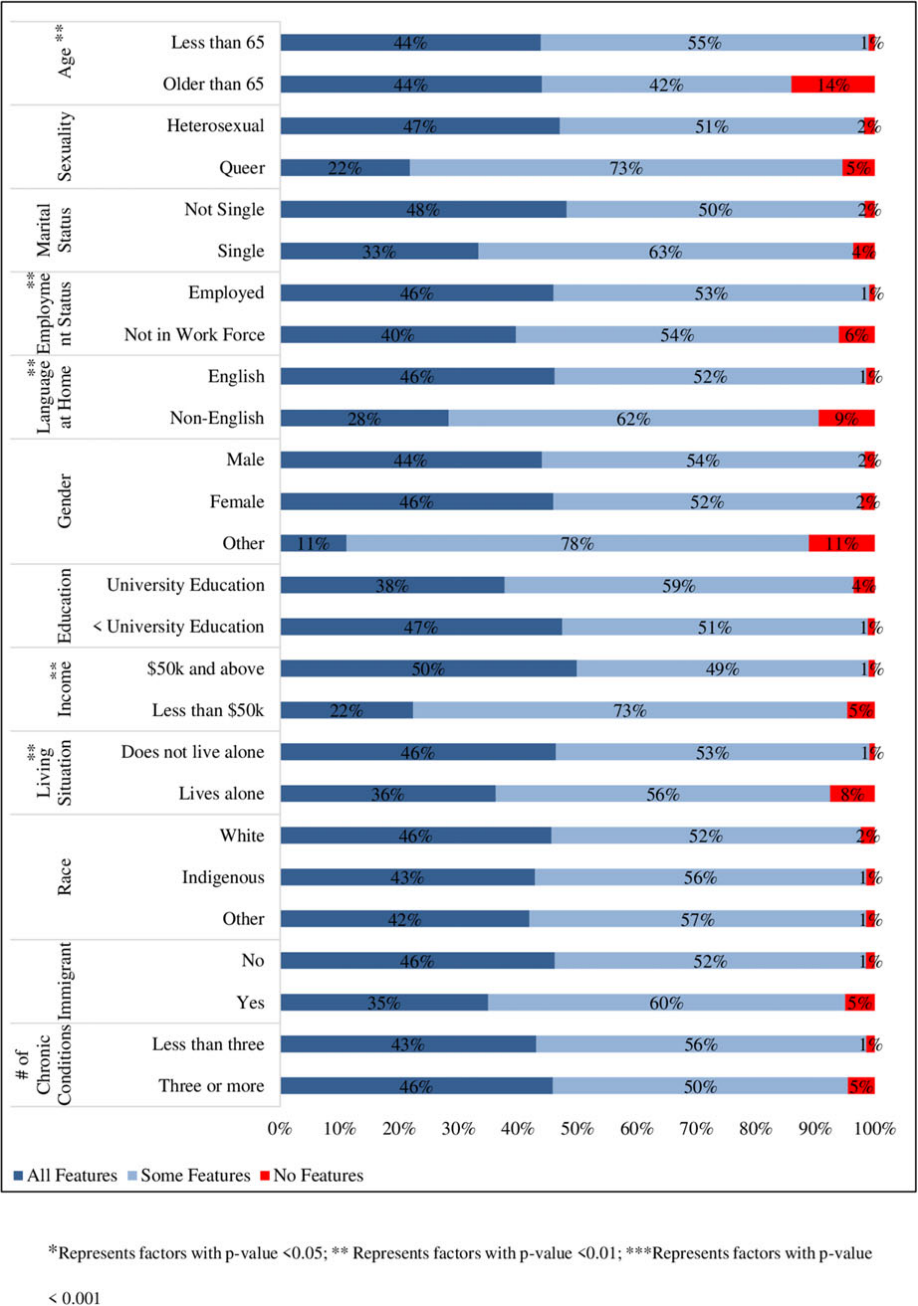
Figure 1 long description. Use of a number of PP features across socio-demographic factors. The values represent the percentages of respondents in each sociodemographic category using all, some, and no features of patient portals.

Some socio-demographic factors were found to be significantly (*p* < 0.05) associated with not using any PP features. The percentages of respondents in the statistically significant socio-demographic categories are as follows: older than 65 years of age (14%) compared to younger than 65 (1%); not in the workforce (6%) compared to employed (1%); using a non-English language at home (9%) compared to speaking English at home (1%); less than $50k income (5%) compared to more than $50k income (1%); and living alone (8%) compared to not living alone (1%). Figure 1 shows these statistically significant factors as well as other socio-demographic factors and the percentages of those who use all, some, and no PP features.

### Sensitivity analysis

In the sensitivity analysis, differences of 10%+ were observed in a few results (Appendix A). Caregivers in the sensitivity analysis had a higher percentage of respondents who were 44 and younger (48%) and recent immigrants (24%), and a lower percentage of respondents who were not employed (39%) compared to respondents in the verified analysis. A higher percentage of patients (72%) and caregivers (75%) in the sensitivity analysis had access to PPs.

## Discussion

Most respondents (patients and caregivers) report having access to PPs in PC in Ontario. As PPs are relatively new in PC in Canada, we expected to have fewer respondents with access to PPs. However, a recent Canadian report supports this as it found that about half of the Canadian population has access to their electronic health information with Ontario having a similar rate of access (StatCAN Plus, 2024). In individuals without PP access, it is possible that a lack of awareness about PP options affects their access.

‘Communicating with the practice’ and ‘viewing chart information’ were identified as the most used and anticipated PP features in patients and caregivers with and without PP access. Previous studies on patient experiences with PP features in PC have similar findings as Casacchia et al. identified secure messaging as the most important PP feature with viewing test results being the second most important (Casacchia *et al*., 2022), and DeJesus et al.’s study found patients had the most experience with viewing test results and messages from their healthcare team (DeJesus *et al*., 2022).

Generally, there was a trend in the use of a number of PP features across socio-demographic factors. Marginalized (*Marginalised Identities – Definition and Explanation*, no date) individuals tended to use all or some PP features at lower percentages and no PP features at higher percentages compared to less marginalized individuals. Respondents who were older than 65, unemployed, who speak a non-English language at home, had less than $50k income or lived alone were found to have significantly higher percentages of individuals indicating not using any PP features. This is consistent with available evidence as studies show that PP users tend to be patients from less marginalized groups such as patients who are more educated, medically insured or have higher income, and tend to comprise of women and younger individuals (El-Toukhy *et al*., 2020; Tossaint-Schoenmakers *et al*., 2021; Casacchia *et al*., 2022). Studies indicate reasons as to why this may be observed in vulnerable populations. As older individuals tend have a preference for in person care (Abdallah *et al*., 2022; Bhatia *et al*., 2022) and tend to have difficulty navigating PP platforms (Fickman, 2023), they tend to be less likely to use PP features. Individuals who are unemployed and have less than $50k income tend to be less likely to use PP features as they may not have the financial resources to purchase technology or deal with internet costs associated with using PPs and their features. Individuals who use a non-English language at home may not use PP features as PPs are typically in English and may not be in languages accessible to them.

### Implications for policy

Although our results are specific to PC, our findings can be applied in other health contexts as well as provide insights into equity aspects to be considered when implementing PPs. As those who are older and speak a non-English language were associated with not using any PP features, we suggest that policy makers responsible for PP implementation ensure that PPs are user friendly, have an interface allowing for easy navigation of PP features on various devices to facilitate access on the device most efficient for use by the patient (e.g. easy interface on laptop, phone and tablet), and are developed in multiple languages to increase accessibility. Unemployed individuals and those who make less than $50k were also found to be associated with not using any PP features. We suggest that PPs should have minimal/no costs associated with their use and access, and that there should be financial incentives to assist with costs associated with accessing and using PPs. Policy makers and organizations which implement PPs should also consider increasing provider incentives for the large-scale adoption of PPs in PC to facilitate virtual care delivery and consider working with marginalized groups during PP development and implementation.

### Limitations

Several study limitations were encountered. The monetary compensation to survey completion and the wide dissemination of the survey allowed bots to penetrate the survey despite the honeypot question included to prevent bot responses. Also, we did not develop measures to prevent multiple survey entries from a single respondent making it difficult to determine if a respondent provided multiple responses to the survey. Future research should consider more efficient bot prevention strategies and establish methods to prevent multiple responses from a single respondent.

As the recruitment approach involved the broad dissemination of the survey through multiple strategies, we did not obtain the number of respondents approached and are unable to determine the survey response rate. The recruitment approach was both a strength and limitation as the study adequately captured the virtual care experiences of individuals in a PC setting. However, the experiences of individuals who could not be reached via social media, advocacy groups or provider networks may not have been captured in the study. Similarly, as the survey was administered digitally, the virtual care experiences of those with limited internet or device access or limited digital literacy may not have been captured in the study. Additionally, the survey was administered in the two official languages (English and French) and may not have captured the experiences of those who speak in non-English or non-French languages. Future research should ensure response rates can be determined and consider optimized recruitment strategies to adequately capture the experiences of all individuals in a population.

As this study is derived from a small section of the larger survey which intended to briefly capture experiences with PPs, the questions do not fully capture all aspects of patient experiences with PPs. Due to this, the survey did not include questions to capture the use of PP features across various PP types or physician payment models and did not include questions to capture possible reasons for not using or not wanting to use PP features limiting our ability to infer causality. Assessing experiences with various PP types and physician payment models would be important to assess the consistency of our results with various PP types and physician payment models and questions which capture reasons for not using or not wanting to use PP features would allow for a more robust interpretation of the findings. Future research should explore these associations, as well as establish questions to adequately capture all aspects of PP feature use.

This study was funded by INSPIRE-PHC. The funding body was not involved in the design of the study nor involvement in the collection, analysis, interpretation of data, or writing of the manuscript.

## Conclusion

This study highlights the use and anticipated use of PP features by Ontario residents in PC. As socio-demographic factors such as age, employment status, income, and language at home were associated with not using any PP features for those with access to PPs, emphasis should be placed on the consideration of these socio-demographic groups during PP development and implementation. PPs should be tailored to the populations where these PPs are to be adopted, implemented, and used.

## Supporting information

10.1017/S1463423626101376.sm001 Aomreore et al. supplementary material 1 Aomreore et al. supplementary material

10.1017/S1463423626101376.sm002 Aomreore et al. supplementary material 2 Aomreore et al. supplementary material

## Data Availability

The data used in this study is available upon request. Please contact the authors to request the data.

## Supplementary material

The supplementary material for this article can be found at https://doi.org/10.1017/S1463423626101376.

## Table 1. Long description

The table compares socio-demographic factors of patients and caregivers across various categories. It includes data on age, gender, sexual orientation, language spoken at home, marital status, education level, employment, household income, immigration status, living situation, race/ethnicity, and Ontario health region. The table has 15 rows and 5 columns, with column headers being Category, Respondents, Patients n (%), Patients n, and Caregivers n (%). Notable trends include a higher percentage of patients under 45 years old compared to caregivers, differences in marital status, education level, and household income. The table provides a detailed comparison of these socio-demographic factors between the two groups.

Navigate back to Table 1..

## Table 2. Long description

A table with five rows and four columns compares the current and anticipated use of patient portal features by patients and caregivers. The columns are labeled ‘Current use of PP features n (%)’, ‘Anticipated use of PP features n (%)’, with sub-columns for ‘Patients’ and ‘Caregivers’. The rows list features: ‘Schedule appointments online’, ‘View chart information’, ‘Enter information into chart’, ‘Communicate with practice’, and ‘Receive documents from practice’. The table shows percentages and counts of patients and caregivers using or anticipating using these features. Notable trends include high current use and anticipated use of communication with practice and scheduling appointments online.

Navigate back to Table 2..

## Figure 1. Long description

The bar graph compares the use of patient portal features across different socio-demographic factors, with horizontal bars representing percentages of respondents using all, some, or no features. The x-axis ranges from zero percentage to one hundred percentage, while the y-axis lists categories such as age, sexuality, marital status, employment status, language spoken at home, gender, education, income, living situation, race, immigrant status, and the number of chronic conditions. Each category is divided into subcategories, with bars color-coded in blue, light blue, and red to indicate the percentage of respondents using all features, some features, and no features, respectively. Notable trends include higher usage of all features among younger individuals, those with university education, and higher income groups. Conversely, certain groups like the elderly, non-English speakers, and those with three or more chronic conditions show a higher percentage of non-usage. All values are approximated.

Navigate back to Figure 1..
