# Supplementary material for: Patient and caregiver use of patient portal features in primary care: a cross-sectional survey study of Ontarians
Source: Prim Health Care Res Dev. 2026 Jun 19;27:e69. doi: 10.1017/S1463423626101376 (PMC13280581; doi:10.1017/S1463423626101376)
Supplement: Aomreore et al. supplementary material 1 — Aomreore et al. supplementary material [file S1463423626101376sup001.docx]

# Appendix A: Sensitivity AnalysEs Results

**Table 1: Sociodemographic Profile of Participants**

|  | **Respondents** | **Patients n (%)** (n=1379) | **Caregivers n (%)** (n=380)^1^ |
| --- | --- | --- | --- |
| **Sociodemographic Factors** | | | |
| Age | 44 and younger | 910 (67) | 168 (48) |
|  | 45 – 65 | 379 (28) | 125 (36) |
|  | 65 and older | 67 (5) | 54 (16) |
| Gender | Male | 686 (51) | 170 (47) |
|  | Female | 643 (47) | 186 (51) |
|  | Other | 25 (2) | 8 (2) |
| Sexual Orientation | Queer | 202 (15) | 29 (8) |
| Language spoken at home | Non-English^2^ | 107 (8) | 43 (12) |
| Marital Status | Single^3^ | 420 (31) | 140 (40) |
| Education Level | Less than a university degree | 738 (55) | 264 (76) |
| Employment | Not employed^4^ | 219 (16) | 139 (39) |
| Household Income | Less than $50k | 373 (29) | 129 (40) |
| Immigration | Recent Immigrant^5^ | 139 (11) | 79 (24) |
| Lives Alone | Yes | 327 (25) | 77 (23) |
| Race/Ethnicity^*^ | Caucasian | 945 (70) | 242 (67) |
|  | Indigenous | 136 (10) | 43 (12) |
|  | Other | 262 (20) | 74 (21) |
| Ontario Health Region | Toronto | 370 (32) | 88 (31) |
|  | Central | 293 (26) | 67 (24) |
|  | East | 181 (16) | 46 (16) |
|  | West | 226 (20) | 67 (24) |
|  | North East | 49 (4) | 10 (4) |
|  | North West | 22 (2) | 4 (1) |
| **Health Related Factors** | | | |
| Chronic Conditions | Mean Number (SD) | 1.5 (1.4) | 2.2 (1.8) |
|  | Three or more conditions (%) | 274 (20) | 142 (38) |

^1^ Represents the profile of the patients on behalf of whom the caregivers responded

^2^ Includes French and other (respondents were able to select more than one language)

^3^Single = never legally married, separated but still legally married, divorced and widowed

^4^Not Working = not employed/looking for work, unable to work due to sickness or disability, retired from paid work, in school, looking after home/family

^5^Recent Immigrant = Individual who immigrated to Canada in past 10 years

^*^Respondents could select more than one option regarding their race. Other includes Black, Arab, Chinese, Filipino, Japanese, Korean, Latin American, South Asian, Southeast Asian, West Asian

**Table 2: Patient Portal Access**

|  | **Patients n (%)**  (n=1377) | **Caregivers n (%)**  (n=378) |
| --- | --- | --- |
| Practice offers patient portal | 985 (72) | 284 (75) |
| Practices does not offer patient portal | 392 (28) | 94 (25) |

**Table 3: Current/Anticipated Use of Features for Respondents with and without Access to PPs**

|  | **Current Use of PP Features (%)**^1^ | | **Anticipated Use of PP Features (%)**^2^ | |
| --- | --- | --- | --- | --- |
|  | Patients  (n=985) | Caregivers  (n=284) | Patients  (n=392) | Caregivers  (n=94) |
| Schedule appointments online | 576 (60) | 197 (77) | 266 (72) | 44 (48) |
| View chart information | 609 (71) | 186 (71) | 270 (72) | 44 (48) |
| Enter information into chart | 562 (67) | 185 (74) | 245 (66) | 50 (54) |
| Communicate with practice | 665 (73) | 214 (81) | 245 (65) | 52 (55) |
| Receive documents from practice | 602 (70) | 206 (78) | 263 (71) | 39 (42) |

^1^ For those with PP access; ^2^ For those without PP access

**Table 4: Current/Anticipated Use of a Number of Patient Portal Features**

|  | **Current Use of PP Features (%)**^1^ | | **Anticipated Use of PP Features (%)**^2^ | |
| --- | --- | --- | --- | --- |
|  | Patients  (n=985) | Caregivers  (n=284) | Patients  (n=392) | Caregivers  (n=94) |
| All features | 380 (39) | 134 (47) | 169 (43) | 17 (18) |
| Some features | 550 (56) | 140 (50) | 182 (47) | 68 (72) |
| None of the features | 47 (5) | 8 (3) | 40 (10) | 9 (10) |

**Table 5: Associations Between the Number of Patient Portal Features Currently Used and Sociodemographic Factors for Patients and Caregivers Combined**

| **Categories** | | **All Features (%)**  (n=514) | **Some Features (%)**  (n=690) | **No Features (%)**  (n=55) |
| --- | --- | --- | --- | --- |
| Age | Less than 65 | 41 | 55 | 4 |
|  | Older than 65 | 40 | 48 | 12 |
| Sexuality | Heterosexual | 43 | 53 | 4 |
|  | Queer | 28 | 68 | 3 |
| Marital Status | Not Single | 44 | 52 | 4 |
|  | Single | 33 | 62 | 6 |
| Employment Status | Employed | 42 | 55 | 4 |
|  | Not in Work Force | 40 | 54 | 6 |
| Language at Home | English | 41 | 55 | 4 |
|  | Non-English | 41 | 53 | 6 |
| Gender | Male | 39 | 55 |  |
|  | Female | 44 | 53 | 3 |
|  | Other | 17 | 75 | 8 |
| Education | University Education | 42 | 54 | 4 |
|  | < University Education | 41 | 55 | 4 |
| Income | $50k and above | 47 | 51 | 2 |
|  | Less than $50k | 26 | 66 | 8 |
| Living Situation | Does not live alone | 45 | 53 | 3 |
|  | Lives alone | 31 | 60 | 9 |
| Race | White | 42 | 53 | 5 |
|  | Indigenous | 42 | 57 | 1 |
|  | Other | 36 | 60 | 4 |
| Immigrant | No | 40 | 57 | 3 |
|  | Yes | 43 | 48 | 9 |
| # of Chronic Conditions | Less than three | 41 | 54 | 5 |
|  | Three or more | 39 | 57 | 4 |
