# Supplementary material for: Patient and caregiver use of patient portal features in primary care: a cross-sectional survey study of Ontarians
Source: Prim Health Care Res Dev. 2026 Jun 19;27:e69. doi: 10.1017/S1463423626101376 (PMC13280581; doi:10.1017/S1463423626101376)
Supplement: Aomreore et al. supplementary material 2 — Aomreore et al. supplementary material [file S1463423626101376sup002.docx]

VCPE Survey – Patient Portals and Sociodemographic Sections

Start of Block: Consent

Q1.1
          
  Consent Information for Recommendations for Patient-Centred Virtual Care   We are inviting Ontario residents to participate in a study to understand their experiences and expectations as a patient in family practices (also called primary care)  
 What is Primary Care Primary care is your main point of contact in the healthcare system. Primary care is concerned with preventing disease; managing new or chronic conditions like an infection, mental health, or asthma; screening for disease like cancer, diabetes, and high blood pressure; and immunization.

 Primary care is usually provided by a family doctor or a nurse practitioner. Primary care practices also commonly have nurses. Some have other healthcare providers like a social worker, pharmacist, dietitian, psychologist, occupational therapist etc.   This Study Over the past 2 years, many primary healthcare appointments took place "virtually" (i.e., by telephone or video), but some were conducted in-person as well. The main purpose of this study is to understand the experiences of Ontario patients and caregivers with the primary care services, and their expectations for the future to establish recommendations to policymakers.
   Study Procedures If you decide to participate, you will complete an **anonymous** 15-to-20-minute online survey that will capture your experience in receiving services in primary care (family practice). After completing the survey, you may provide your contact information (optional) to receive a $5 gift card and enter into a draw for a $100 gift card as a small recognition of your contribution. That contact information will be stored separate from the survey responses which means that your survey responses will remain anonymous.
   Risks and Voluntary Participation There are no physical or psychological/emotional risks expected. You may experience minor discomfort from sharing any challenges that you have faced related to your care. Participation in this study is voluntary and you may choose to leave questions unanswered. You can discontinue your participation at any time.
   Benefits Individual participants will not directly benefit from the study. However, your participation will help develop recommendations that may influence the future of virtual care in primary care. The recommendations from this study could lead to changes in health care services that benefit individuals across Ontario accessing care virtually.
   Confidentiality Your participation and responses will be confidential. Our research team are the only individuals who will have access to your survey responses. They will summarize the survey data so it can be shared through research reports and presentations without any possibility of identifying participants. In addition, the personal information you provide to allow us to give you a $5 gift card and enter you into a draw for a $100 gift card will be removed from the survey data before it is analyzed. This survey is created using a software program called Qualtrics, which collects and securely stores and backs up survey data in Toronto, Ontario and Montreal, Quebec. The anonymous survey data will be kept on a password-protected computer database at the University of Toronto and the University of Ottawa and will be permanently deleted after seven years.

 This study is led by Dr. Rachelle Ashcroft (Factor-Inwentash Faculty of Social Work, University of Toronto) and Dr. Simone Dahrouge (Department of Family Medicine, University of Ottawa). Should you have any questions, please contact Rachelle Ashcroft at [rachelle.ashcroft@utoronto.ca](mailto:rachelle.ashcroft@utoronto.ca).

This study has been reviewed and received ethics clearance through a University of Toronto Research Ethics Board (RIS #43446) and the Bruyère Research Institute (#M16-22-054). If you have any comments or concerns from participating in this study, please contact Daniel Gyewu in the Office of Research Ethics at 416-978-3165 or at [dgyewu@utoronto.ca](mailto:dgyewu@utoronto.ca) in Toronto, or the Bruyère Research Office in Ottawa at (613) 562-6262 Ext. 4003 or [REB@bruyere.org](mailto:REB@bruyere.org).Please note that this study is evaluating only the care you received in **primary care (family practice)**. It **does not concern specialty care** that surgeons, oncologists, endocrinologists, and other specialists provide or care you receive in an emergency room.

**Please complete this survey only if you receive primary healthcare services in Ontario.**

Q1.2 Do you wish to complete the survey?

- Yes, I understand the information provided and wish to complete the survey (1)
- No, I do not wish to complete the survey (2)

Skip To: End of Survey If Q1.2 = 2

Q1.3 Please check the box below to continue with the survey

End of Block: Consent

Q9.8 Some practices offer patients the ability to use a secure platform that allows them to, for example, view the results of their laboratory tests, request a prescription renewal, receive notification from their provider and send messages to their provider. These platforms are called **patient portals**.

Q9.9 Does your Primary Care Practice offer a patient portal?

- Yes, my practice offers an online patient portal (1)
- No, my practice does not offer an online patient portal (2)
- I do not know if my practice offers an online patient portal (3)

Display This Question:

If Q9.6 = 1

Q9.10 Please share if you use your practice's online patient portal to access the following features:

|  | I use this feature (1) | I do not use this feature (2) | Not offered or I'm not sure (3) |
| --- | --- | --- | --- |
| Communicate with my practice (ask questions, receive messages from my providers) (1) |  |  |  |
| View my chart information (e.g. bloodwork and test results) (2) |  |  |  |
| Enter information into my chart (e.g. my blood pressure) (3) |  |  |  |
| Receive documents from my doctor (e.g. prescription or medication information) (4) |  |  |  |
| Schedule my appointments online (5) |  |  |  |
| Other; please specify: (6) |  |  |  |

Display This Question:

If Q9.6 = 2

Or Q9.6 = 3

Q9.11 Please share if the following features would be useful to you in an online patient portal:

|  | I would use this feature (1) | I don't need this feature (2) | I'm not sure (3) |
| --- | --- | --- | --- |
| Communicate with my practice (ask questions, receive messages from my providers) (1) |  |  |  |
| View my chart information (e.g. bloodwork and test results) (2) |  |  |  |
| Enter information into my chart (e.g. my blood pressure) (3) |  |  |  |
| Receive documents from my doctor (e.g. prescription or medication information) (4) |  |  |  |
| Schedule my appointments online (5) |  |  |  |
| Other; please specify: (6) |  |  |  |

Q9.12 Please share with us any additional recommendations you have on the future of telephone and/or video appointments

________________________________________________________________

________________________________________________________________

________________________________________________________________

________________________________________________________________

Q11.2 Please indicate if you have any of the following conditions if they have lasted longer than 6 months, have been confirmed by a doctor or nurse, or for which you are taking prescribed medication. (check all that apply)

- High blood pressure (Hypertension) (1)
- High cholesterol (Hyperlipidemia) (2)
- Arthritis (Rheumatoid or osteoarthritis) (3)
- Persistent back/sciatic pain or persistent joint and muscle pain (non-arthritis) (4)
- Stomach problem (Reflux, ulcer or heartburn) (5)
- Depression or anxiety (6)
- Thyroid disorder (7)
- Asthma, chronic obstructive pulmonary disease (COPD) or chronic bronchitis (8)
- Diabetes (9)
- Heart problem (angina, myocardial infarction, atrial fibrillation, poor circulation in lower limb) (10)
- Bowel/Colon problem (irritable bowel, Crohn’s disease, ulcerative colitis, diverticulosis) (11)
- Other, please specify: (12) __________________________________________________
- ⊗None of the above mentioned (13)

Skip To: End of Block If Q11.2 = 13

Skip To: End of Block If Condition: Selected Count Is Equal to 0. Skip To: End of Block

End of Block: Health Conditions

Start of Block: Demographics

Q12.1 Getting to Know You In this section, we ask you to provide some information about yourself to allow us to understand the needs and expectations of those with diverse backgrounds and from locations across Ontario. Please be assured that all this information is **confidential** and **will not be shared with anyone**. Only summary, anonymous data will be made public.

Q12.2 What is your age (in years)?

- 18 to 24 (1)
- 25 to 34 (2)
- 35 to 44 (3)
- 45 to 54 (4)
- 55 to 64 (5)
- 65 to 74 (6)
- 75 to 84 (7)
- 85 and over (8)
- Prefer not to answer (9)

Q12.3 What is your gender identity?

- Male (1)
- Female (2)
- Non-binary (3)
- Trans male (4)
- Trans female (5)
- Two-Spirit (6)
- Other, please specify: (7) __________________________________________________
- Prefer not to answer (8)

Q12.4 What is your sexual orientation?

- Heterosexual/Straight (1)
- Gay (2)
- Lesbian (3)
- Bisexual (4)
- Queer (5)
- Pansexual (6)
- Asexual (7)
- Other, please specify: (8) __________________________________________________
- Prefer not to answer (9)

Q12.5 What is your race and/or ethnicity? (check all that apply)

- Arab (17)
- Black (6)
- Chinese (11)
- Filipino (5)
- First Nations (21)
- Inuk/Inuit (23)
- Japanese (19)
- Korean (18)
- Latin American (12)
- Métis (22)
- South Asian (e.g., East Indian, Pakistani, Sri Lankan) (13)
- Southeast Asian (e.g., Vietnamese, Cambodian, Laotian, Thai) (14)
- West Asian (e.g., Iranian, Afghan) (9)
- White/Caucasian (15)
- Other, please specify: (16) __________________________________________________
- ⊗Prefer not to answer (20)

Q12.6 What language(s) do you usually speak at home? (check all that apply)

- English (1)
- French (2)
- Other, please specify: (3) __________________________________________________
- Prefer not to answer (4)

Q12.7 In which of Canada's official languages do you prefer receiving healthcare services?

- English (1)
- French (2)
- Prefer not to answer (3)

| Page Break |  |
| --- | --- |

Q12.8 What is your marital status?

- Never legally married (1)
- Legally married or common-law (and not separated) (2)
- Separated, but still legally married (3)
- Divorced (4)
- Widowed (5)
- Other, please specify: (6) __________________________________________________
- Prefer not to answer (7)

Q12.9 What is the highest level of education you have completed?

- Some secondary or higher school, not completed (1)
- High school completed (2)
- Had some university education or completed a community college, technical college, or post-secondary program (e.g., CEGEP, trade, vocational) (3)
- Completed a bachelor's degree (e.g., BA, BSc, BSN) (4)
- Completed a graduate or professional degree (e.g., Master's, MBA, MD, PhD) (5)
- Other, please specify (6) __________________________________________________
- Prefer not to answer (7)

Q12.10 What is your current employment status? (if more than one of these applies to you, **check the main ONE only**)

- Employed full time (30 hours or more each week) (1)
- Employed part time (Less than 30 hours each week) (2)
- Unemployed and looking for work (3)
- In school (4)
- Unable to work due to sickness or disability (5)
- Looking after home/family (6)
- Retired from paid work (7)
- Other, please specify: (8) __________________________________________________
- Prefer not to answer (9)

Q12.11 What is your household income?

- No income (1)
- $1 to $24,999 (2)
- $25,000 to $49,999 (3)
- $50,000 to $99,999 (4)
- $100,000 to $149,999 (5)
- $150,000 to $199,999 (6)
- $200,000 and over (7)
- Prefer not to answer (8)

| Page Break |  |
| --- | --- |

Q12.12 Did you immigrate to Canada in the last 10 years?

- Yes (1)
- No, I immigrated more than 10 years ago (2)
- No, I am a Canadian citizen by birth (3)
- Prefer not to answer (4)

Q12.13 Do you live alone?

- Yes (1)
- No (2)
- Prefer not to answer (3)

Q12.14 Please share the name of the city or town where you currently reside:

________________________________________________________________

| 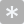 |
| --- |

Q12.15 What is your postal code?

________________________________________________________________

End of Block: Demographics
